# Supplementary material for: Time derivatives via interconnected waveguides
Source: Sci Rep. 2023 Aug 12;13:13126. doi: 10.1038/s41598-023-40046-3 (PMC10423277; doi:10.1038/s41598-023-40046-3)
Supplement: Supplementary file 1 — Supplementary Information. [file 41598_2023_40046_MOESM1_ESM.pdf]

Supplementary information

Time derivatives via interconnected waveguides

Ross Glyn MacDonald[1,2]*, Alex Yakovlev*[2] *and Victor Pacheco-Peña*[1*]

[1]*School of Mathematics, Statistics and Physics, Newcastle University, Newcastle Upon Tyne, NE1 7RU, United Kingdom*
[2]*School of Engineering, Newcastle University, Newcastle Upon Tyne, NE1 7RU, United Kingdom*
**email:* victor.pacheco-pena@newcastle.ac.uk

1. **Generalized *M*-stub transfer function.**
2. **First order differentiators from series junctions and open stubs, reflection and transmission mode**
3. **Effect of length of connection between junctions: cascaded temporal differentiator performance**
4. **First order differentiator: bandwidth study**

1. Generalized M-stub transfer function

Here we derive the transmission coefficient of an arbitrary junction of waveguides consisting of $N = M + 2$ waveguides connected at a junction in either series or parallel configuration: two waveguides, one as input and one as output, and M-stubs. As in the main text, the input and output waveguides are identical with the same characteristic impedance $Z_0 = (h_0/w_0)\eta_0$, where h_0, w_0 is the waveguide separation and width, respectively, as shown in Fig. 1 of the main text, and $\eta_0 = \sqrt{\mu_0/\varepsilon_0}$ is the impedance of free space (i.e. input and output waveguides are filled with vacuum $\mu_r = \varepsilon_r = 1$). The length of the stubs is defined as L_{sj} (with "s" as stub and $j = 1, 2, 3, \ldots, M$ is the stub label used to distinguish between the various stubs connected at the junction) and the reflection coefficient of the stubs as $\Gamma_{j,\pm 1}$, (where $+1$ and -1 refers to an open and closed stub respectively). The characteristic impedance of the connected stubs is defined as $Z_{sj} = (h_{sj}/w_{sj})\eta_{sj}$, with $\eta_{sj} = \eta_0\sqrt{\mu_{rj}/\varepsilon_{rj}}$, with μ_{rj} and ε_{rj} as the relative permeability and permittivity of the stub waveguide j, respectively.

With this configuration, the splitting and superposition of signals at the junction between such multiple waveguides can be described as a scattering matrix $\boldsymbol{A}$ [of size N by N], with $\boldsymbol{y} = \boldsymbol{A}\boldsymbol{x}^T$. In this expression $\boldsymbol{x} = [x_1, x_2, \ldots, x_N]$ and $\boldsymbol{y} = [y_1, y_2, \ldots, y_N]^T$ are vectors containing the amplitudes of the incident (from all the waveguides towards the junction) and outgoing signals (scattered from the junction). The superscript T is the transpose operator. Note that the matrix $\boldsymbol{A}$ can be defined for a junction of connected waveguides in series ($\boldsymbol{A}_{series}$) or parallel ($\boldsymbol{A}_{parallel}$) configuration, as shown in Eq. 1 from the main text. For the sake of simplicity, here we call it as $\boldsymbol{A}$ to avoid rewriting all the expressions in terms of series or parallel configuration. With this in mind, the elements of the matrix $\boldsymbol{A}$, representing the scattering at a single isolated junction can be defined for the parallel (Eq. S1a) and series (Eq. S1b) junctions, as follows:

$$A_{o,(i\neq o)} = \frac{2}{Z_i \sum_{b=1}^{M+2} \frac{1}{Z_b}}, \quad A_{o,(i=o)} = \left(\frac{2}{Z_i \sum_{b=1}^{M+2} \frac{1}{Z_b}} - 1\right) \tag{S1a}$$

$$A_{o,(i\neq o)} = \frac{-2Z_i}{\sum_{b=1}^{M+2} Z_b}, \quad A_{o,(i=o)} = \left(1 - \frac{2Z_i}{\sum_{b=1}^{M+2} Z_b}\right) \tag{S1b}$$

where the left and right terms in Eq. S1a,b represent the off-diagonal and diagonal term of matrix $\boldsymbol{A}$, respectively, $A_{o,i}$ describes the scattering towards waveguide o from waveguide i, and Z_i refers to the characteristic impedance of waveguide i connected to the junction. Here o and $i = 1, 2, 3, \ldots, N$ are the waveguide labels of all the connected waveguides (this includes the input, output, and stub waveguides). Note that when all impedances in Eq. S1 are the same, the off-diagonal and diagonal terms converge into the matrix elements shown in Ref. 16 from the main text. In our work, the input and output waveguides are labeled as $M + 1$ and $M + 2$,

respectively. This means that the magnitude of the *first split* observed at the output waveguide, as described in the main text, is given by $A_{M+2,M+1}x_{in}$ (as mentioned above as $\boldsymbol{y} = \boldsymbol{A}\boldsymbol{x}^T$), where x_{in} is the incident signal applied at the input waveguide (waveguide $M + 1$).

As is described in the main text, the interaction of an incident signal at the junction may be thought of as the superposition of two splitting events, called the *first* and *second* split. During the *first split* a portion of the incident signal x_{in} is transmitted to each of the connected waveguides (including stubs, input, and output waveguides). In this realm, outward traveling signals $y_j = A_{j,M+1}x_{in}$, $y_{M+2} = A_{M+2,M+1}x_{in}$ and $y_{M+1} = A_{M+1,M+1}x_{in}$ are produced in the stubs, output waveguide and input waveguide respectively, from the junction. The signals traveling within the stubs are then reflected by the open/closed ends of the stubs redirecting them back into the junction. Once these signals reach and pass the junction, they will produce a *second split*, as explained in the main text, with a time delay between the *first* and *second splits* due to the travel time of the signals within the stubs of $\Delta t = 2L_{sj}\sqrt{\varepsilon_{rj}\mu_{rj}}/c$, where c is the speed of light in a vacuum,. As explained in the main text, assuming the incident signal is temporally long compared to Δt, when the inward traveling signals arrive at the junction and produce the second split, the incident signal x_{in} is still being applied at the input waveguide. This incident signal will also split at the waveguide junction producing a *new first split*, which will in turn lead to a *new second split* and so on, until the incident signal is no longer applied (when considering modulated broadband incident signals). Because of this performance, the total signal observed at the input (y_{M+1}) and output (y_{M+2}) waveguides, at any given time, is a superposition of the signals transferred by both the *first* and *second splits*, mathematically defined as follow:

$$y_{M+2} = A_{M+2,M+1}x_{in} + \sum_{j=1}^{M} A_{M+2,j}x_j \tag{S2a}$$

$$y_{M+1} = A_{M+1,M+1}x_{in} + \sum_{j=1}^{M} A_{M+1,j}x_j \tag{S2b}$$

where x_j are the elements of $\boldsymbol{x}$ which contain the signals traveling towards the junction (inward signals) from the stub waveguides due to the reflection at the end of the stubs and $A_{M+2,j}$ are the scattering coefficients from the stub waveguides to the output waveguide (column $M + 2$, row elements from 1 to M). The first and second terms of Eq. S2 represent the signal produced by the *first* and *second split* respectively.

The inward traveling signals (reflected signals at the end of the stubs which are responsible for the *second split* after they pass the junction) within the stubs x_j can be related to the outward traveling signals from the junction (produced by the *first split*) y_j by considering the reflection coefficient and phase change of each individual stub as follows:

$$x_j = \Gamma_{j,\pm 1} e^{-2i\varphi_j} y_j \tag{S3}$$

where $\varphi_j = \omega L_{sj}\sqrt{\varepsilon_{r,j}\mu_{r,j}}/c$ is the electrical length of the stubs, $\varepsilon_{r,j}$ and $\mu_{r,j}$ are the relative permittivity and permeability of the filling material of the stubs, respectively.

It is important to note that, in general, the signals involved in the *second split* (i.e., those reflected at the end of the stubs that travel towards the junction) will be scattered between all connected waveguides including the stub waveguides when considering different parameters of L_{sj}, Z_{sj} and $\Gamma_{j,\pm 1}$ for each stub. With this in mind, the outgoing signals within the stubs, from the junction, can be written as a simple superposition of the signals produced by the *first* and *second splits*. This is similar to the signals in Eq. S2, but now selecting the matrix elements of $\boldsymbol{A}$ to represent the scattering towards the stubs instead, we obtain:

$$y_j = A_{j,M+1} x_{in} + \sum_{k=1}^{M} A_{j,k} x_k \tag{S4}$$

where the left and right terms represent the *first* and *second split* respectively, $A_{j,k}$ are the elements of $\boldsymbol{A}$ which describe the scattering of signals between stubs and x_k for $k = 1, 2, 3, \dots, M$ is the signal traveling towards the junction from stub k. Note that the case described in the main text (Fig. 3) is a special scenario when two identical stubs (same L_{sj}, Z_{sj} and $\Gamma_{j,\pm 1}$) are connected at the junction. In this latter case, the superposition of the scattered signals produced by the *second split*, traveling within each stub will destructively interfere, producing no signals within the stubs after this *second split.* This means that, the only the signal that will now enter the stubs will come from the *new first split* produced by the new portion of the incoming incident wave (i.e. the summation operation in the right term of Eq. S4 becomes zero).

Combining Eq. S4 with Eq. S3 gives an expression relating the outgoing signals y_j to one another and the incident signal x_{in}.

$$y_j = A_{j,M+1} x_{in} + \sum_{k=1}^{M} A_{j,k} \Gamma_k e^{-2i\varphi_k} y_k \tag{S5}$$

Eq. S5 can be rearranged and solved for y_j to express the outgoing signals in terms of the parameters ($\boldsymbol{A}$, Γ_{sj} and φ_{sj}) and the incident signal only. To do this, we define a matrix $\boldsymbol{C}$ which relates the outgoing to the inward traveling signals, within the stubs, such that:

$$\sum_{k=1}^{M} C_{j,k} y_k = A_{j,M+1} x_{in} \tag{S6a}$$

$$C_{j,k} = \delta_{j,k} - A_{j,k} \Gamma_{k\pm 1} e^{-2i\varphi_k} \tag{S6b}$$

Substituting $\boldsymbol{C}$, as defined in Eq. 6b, into Eq. S5 and solving for y_j gives:

$$y_j = \sum_{k=1}^{M} (\boldsymbol{C})_{j,k}^{-1} A_{k,M+1} x_{in} \tag{S7}$$

where $(\boldsymbol{C})_{j,k}^{-1}$ is the matrix inverse operation applied to the matrix $\boldsymbol{C}$. Combining Eq. S7, S3 and S2 yields the

signal observed at the input and output waveguides

$$y_{M+2} = [A_{M+2,M+1} + \sum_{j=1}^{M}\sum_{k=1}^{M} A_{M+2,j}\Gamma_{j,\pm1}e^{-2i\varphi_j}(\boldsymbol{C})^{-1}_{j,k}A_{k,M+1}]x_{in} \quad \text{(S8a)}$$

$$y_{M+1} = [A_{M+1,M+1} + \sum_{j=1}^{M}\sum_{k=1}^{M} A_{M+1,j}\Gamma_{j,\pm1}e^{-2i\varphi_j}(\boldsymbol{C})^{-1}_{j,k}A_{k,M+1}]x_{in} \quad \text{(S8a)}$$

with the transmission (Eq. S9a) and reflection (Eq. S9b) coefficients of the overall structure as:

$$T = \frac{y_{M+2}}{x_{in}} = [A_{M+2,M+1} + \sum_{j=1}^{M}\sum_{k=1}^{M} A_{M+2,j}\Gamma_{j,\pm1}e^{-2i\varphi_j}(\boldsymbol{C})^{-1}_{j,k}A_{k,M+1}] \quad \text{(S9a)}$$

$$\Gamma_{M+1} = \frac{y_{M+1}}{x_{in}} = [A_{M+1,M+1} + \sum_{j=1}^{M}\sum_{k=1}^{M} A_{M+1,j}\Gamma_{j,\pm1}e^{-2i\varphi_j}(\boldsymbol{C})^{-1}_{j,k}A_{k,M+1}] \quad \text{(S9b)}$$

These general expressions can be applied to any possible designs; however, this can be simplified by restricting design space such that symmetries of the structure may be exploited. For example, by fixing the impedance of the stub waveguides to be $Z_{sj} = Z_0$ (i.e. all waveguides have the same characteristic impedance, free space impedance in this case) the scattering matrix can be written as: $\boldsymbol{A}_{parallel} = -\boldsymbol{I} + \gamma\boldsymbol{J}$ and $\boldsymbol{A}_{series} = \boldsymbol{I} - \gamma\boldsymbol{J}$ for parallel and series junctions respectively, where $\gamma = 2/N$ and $N = M + 2$ is the total number of waveguides connected at the junction, $\boldsymbol{I}$ and $\boldsymbol{J}$ are the identity and all one matrix, respectively, which corresponds to Eq. 1 shown in the main text of the manuscript and in agreement with Refs. 16 and 18 from the main text.

With this simplification in mind, Eq. S9 may be rewritten using $A_{o,i} = \phi[2/(M+2) - \delta_{o,i}]$, where ϕ is a variable which has been introduced to track the sign change between the series and parallel junctions as $\boldsymbol{A}_{parallel} = -\boldsymbol{A}_{series}$ ($\phi = 1$ and $\phi = -1$ for parallel and series junctions, respectively). Substituting these expressions into Eq. S9 gives the simplified transmission and reflection coefficients, as follow:

$$T = \frac{2\phi}{M+2} + \frac{4}{(2+M)^2}\sum_{j=1}^{M}\sum_{k=1}^{M}\Gamma_{j,\pm1}e^{-2i\varphi_j}(\boldsymbol{C})^{-1}_{j,k} \quad \text{(S10a)}$$

$$\Gamma_{M+1} = \frac{2\phi}{M+2} - \phi + \frac{4}{(2+M)^2}\sum_{j=1}^{M}\sum_{k=1}^{M}\Gamma_{j,\pm1}e^{-2i\varphi_j}(\boldsymbol{C})^{-1}_{j,k} \quad \text{(S10b)}$$

with

$$C_{j,k} = \delta_{j,k} - \frac{2\phi}{M+2}\Gamma_{k,\pm1}e^{-2i\varphi_k} \quad \text{(S10c)}$$

Further simplifications can be made if the open/closed nature and electrical length of all stub waveguides is the same (i.e. $\Gamma_j = \Gamma$ and $\varphi_j = \varphi_s$ in Eq. S3b, where Γ and φ_s is the reflection coefficient and electrical length of all stub waveguides j). Furthermore, due to the assumed symmetry of the system, when considering perfect splitting, it is expected that the outgoing signals (transferred into the stubs from the *first split*) and inward traveling signals (reflected into the junction and responsible for the *second split*) in the stubs will be the same for

all stubs such that $x_j = x_s$ and $y_j = y_s$ for all j. Considering this Eq. S3 can be rewritten for this simplified case as:

$$x_s = \Gamma e^{-2i\varphi_s} y_s \tag{S10}$$

which allows Eq. S3, S5 and S6 to be rewritten in the simplified form, as follows:

$$y_{M+2} = \frac{2\phi}{2+M}(x_{in} + Mx_s) \tag{S11a}$$

$$y_{M+1} = \frac{2\phi}{2+M}(x_{in} + Mx_s) - \phi \tag{S11b}$$

$$y_s = \frac{2\phi}{2+M}[x_{in} + (M-1)x_s] - \frac{\phi M}{M+2}x_s \tag{S11c}$$

$$y_s = \frac{2\phi x_{in}}{M+2-(M-2)\Gamma e^{-2i\varphi_s}} \tag{S11d}$$

Finally, by combining the expressions of Eq. 11 the simplified transmission and reflection coefficients of the structure are written as:

$$T = \frac{2\phi}{M+2}[1 + \phi\frac{2M\Gamma e^{-2i\varphi_s}}{M+2-(M+2)\Gamma e^{-2i\varphi_s}}] \tag{S12a}$$

$$\Gamma_{M+1} = \frac{2\phi}{M+2}[1 + \phi\frac{2M\Gamma e^{-2i\varphi_s}}{M+2-(M+2)\Gamma e^{-2i\varphi_s}}] - 1 \tag{S12b}$$

where Eq. S12a corresponds to the equation shown in Eq. 4 of the main text when $\phi = 1$ (i.e. a parallel junction).

2. First order differentiators from series junctions and open stubs, reflection, and transmission mode

In the main text, we discussed the analytically calculated transfer functions of *M*-stub parallel junctions with stubs terminated with Perfect Electric conductors (PEC). However, it is also possible to perform differentiation of temporal signals by either using the reflection spectrum of the waveguide junctions or by exploiting open-ended stubs. These two cases are this discussed in this section and are shown in Fig. S1. As in Fig. 1 of the main text, the reflection and transmission coefficients of a parallel junction are calculated using Eq. S12 when 1 to 5 stubs are connected at the junction. As in the example provided in Fig. 1c of the main text, all stub lengths in the calculation are chosen to be $L_s = \lambda_0/2$. This was done to better compare the results of the calculated transmission/reflection coefficients and the results shown in Fig. 1c of the main text. With this setup, Fig S1a,b shows the same scenario as presented in Fig. 1 of the main text (PEC terminated stubs) but when working in reflection configuration (amplitude and phase of the reflection coefficient). For completeness, the transmission and reflection spectra are also calculated when using open-ended stubs and the results are shown in Fig. S1d and Fig. S1f respectively. As observed, the results from Fig. S1a,b and Fig. S1e,f are similar, with the former working in reflection and the later in transmission, respectively. These results are as expected because of the flip in sign of the reflected signal from the stubs (signal which produces the *second split*), between the PEC-ended and open-ended cases.

As shown in Fig. S1b, the linear and symmetric dip of the reflection coefficient occurs at $f = f_0/2$ and $f = 3f_0/2$ corresponding to a stub length of $L_s = \lambda/4$ and $L_s = 3\lambda/4$ respectively (λ is the wavelength corresponding to the frequency at which the minimum appears, $2\lambda_0$ and $2\lambda_0/3$ in these cases). In this example the maximum bandwidth of the linear region is achieved when a single stub is connected to the waveguide junction. Fig S1d shows the reflection spectra in the scenario when open-ended stubs are connected at the junction. In this case, the linear dip in the reflection coefficient occurs at $f = 0$, $f = f_0$ and $f = 2f_0$ corresponding to stub lengths which are even integer multiples of $\lambda/2$, again with λ as the wavelength where a dip appears. As in the example shown in Fig. 1 of the main text, the existence of a minimum at $f = 0$ implies that this structure may also be used to perform differentiation onto unmodulated incident signals. These minima are at the same frequencies as the example presented in Fig. 1c, however now the bandwidth is maximized when 1 stub is attached at the waveguide junction instead of 3 as seen in Fig. 1c of the main text. This is an expected result when operating in reflection configuration, as the reflection and transmission coefficients are complementary, a feature that can be clearly seen in Fig. S1c,d and Fig. S1e,f. For completeness, the transmission coefficient for the scenario

discussed in Fig. S1c is presented in Fig. S1f, where the minima appear at the same frequencies at the example given in Fig. S1b, as described above. As in Fig. 1 of the main text, the bandwidth is maximized when 3 stubs are connected at the waveguide junction.

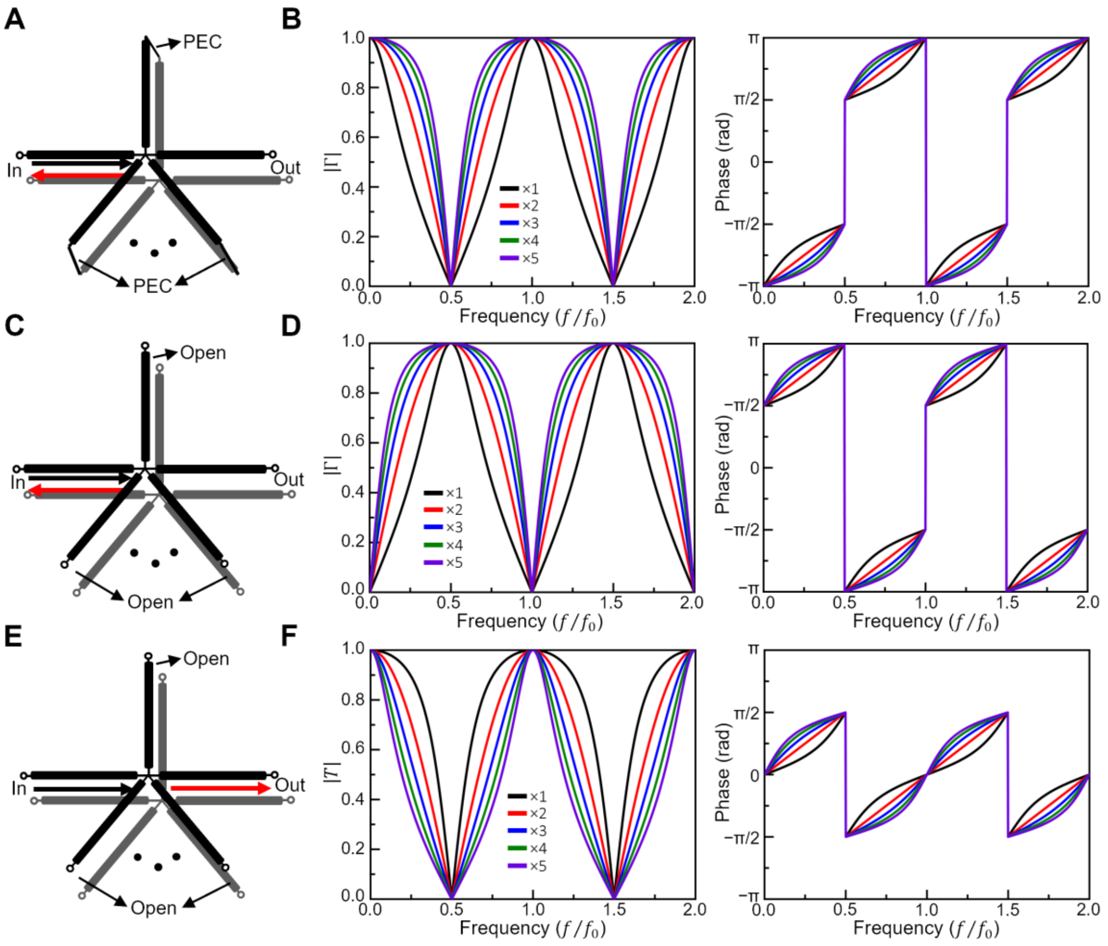

Fig S1| Parallel junction of waveguides: analytical results of their corresponding transfer functions. a, **c**, and **e**, show the transmission line (TL) schematic representation of M stubs connected to an input and output waveguide at a parallel junction. The incident signal is applied from the left waveguide (black arrow). The output signal is recorded either from the left or right waveguides when working in reflection or transmission configuration, respectively (red arrow). **a**, A junction working in reflection using PEC terminated stubs. **b**, A junction working in reflection using open-ended stubs. **c**, A junction working in transmission using open-ended stubs. **b**, **d**, and **f**, reflection/transmission spectra (magnitude, left, and phase, right panels) for the scenarios presented in **a**, **c**, and **e**, respectively, using M stubs from 1 to 5 (black, red, blue, green and purple lines, respectively).

The examples shown in Fig. S1 and in Fig. 1 from the main text demonstrate the use of four possible setups associated with parallel junctions. However, as mentioned in the main text, it is also possible to exploit series junctions with either PEC or open-ended stubs to provide the required transmission and reflection spectra for the differentiation of temporal signals. The four possible setups are presented in Fig. S2 where it can be observed how the four spectra share properties with the parallel case, as expected. In each case, the spectra for the series junction configuration with open-ended stubs is identical to the spectra in a parallel junction with closed

stubs and vice versa. This is expected as a transmission and reflection coefficients of the series and parallel configurations have different signs (see Ref. 16 from the main text). Because of this, the signals reflected from the PEC/open-ended stubs will have different sign when reaching the junction for the series configuration (compared to the parallel scenario) producing complementary performances to the cases discussed in the parallel junctions.

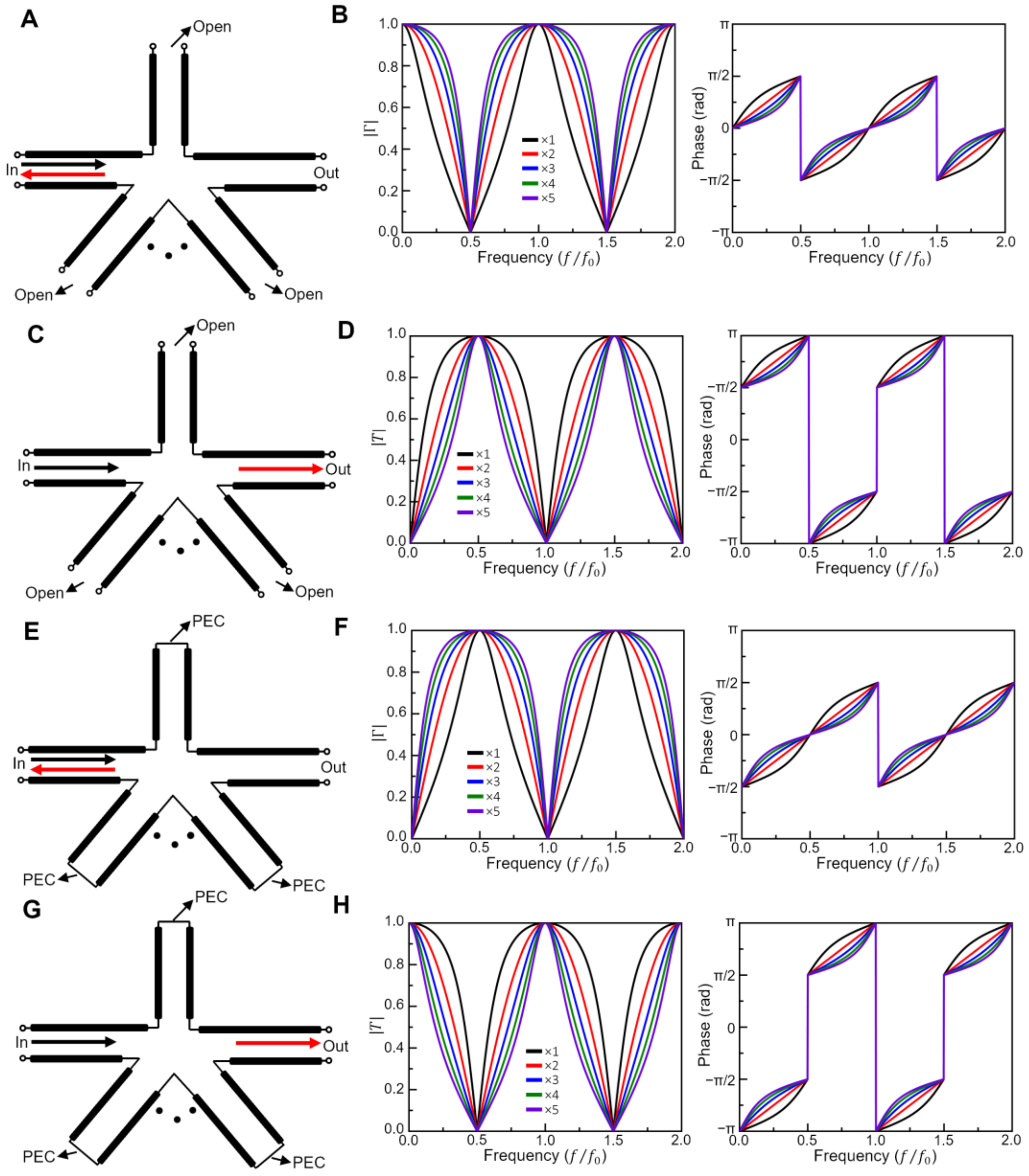

Fig S2| Series junction of waveguides: analytic results of their transfer functions. a, **c**, **e**, and **g**, TL schematic representations of M stubs connected to an input and output waveguide at a series junction. As in Fig. S1, an incident signal is applied from the left waveguide (black arrow) and the output signal (red arrow) is recorded from either the left or right waveguide, when working in reflection or transmission configurations respectively. **a**, A junction working in reflection using open-ended stubs. **b**, A junction working in transmission using open-ended stubs. **c**, A junction working in reflection using PEC terminated stubs. **d**, A junction working in transmission using PEC terminated stubs. **b**, **d**, **f**, and **h**, reflections/transmission spectra (magnitude, left, and phase, right panels) for the scenarios presented in **a**, **c**, **e**, and **g**, respectively, using M stubs from 1 to 5 (black, red, blue, green and purple lines, respectively).

3. Effect of length of connection between junctions: cascaded temporal differentiator performance

As discussed in the main text, temporal differentiators may be cascaded together to perform higher order temporal differentiation by using a connecting waveguide of length L_c between them. In general, due to the large reflection coefficient of a differentiator when working in transmission configuration (considering neglecting losses), a large standing wave will be produced between differentiating "blocks". A schematic representation of this is scenario is presented in Fig. S3a, showing how, the multiple reflections produced between differentiators can interfere with the signal transmitted through the combined structure such that the observed output will not necessarily resemble the second order derivative of the incident signal, as explained in the main text. As is known from TL theory, this interference can be compensated by choosing the length of connecting waveguide such that the multiple reflections between the blocks destructively interfere with each other and thus do not impact the output signal.

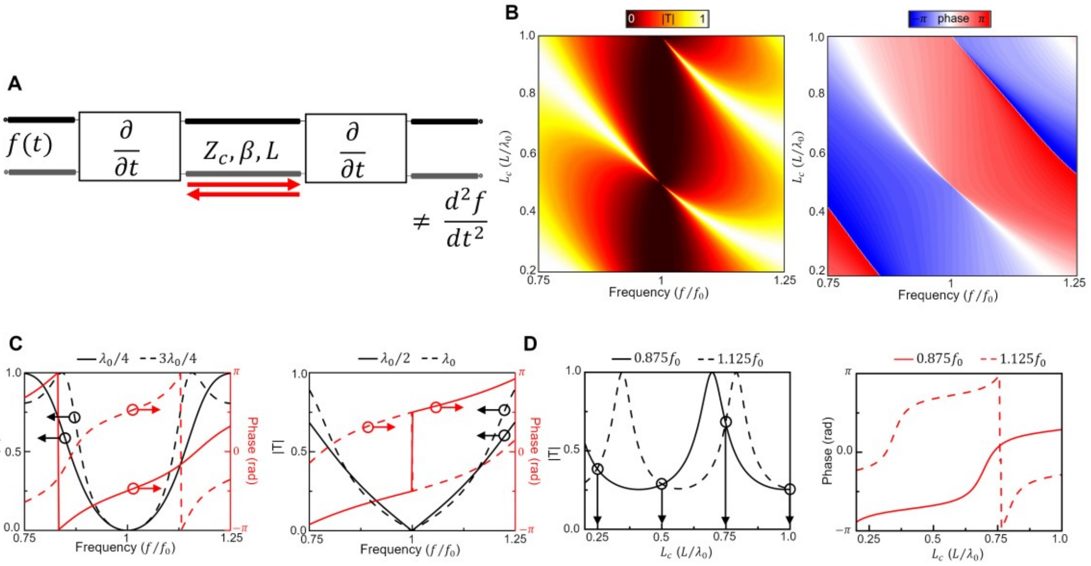

Fig S3| Cascaded temporal differentiator. a, TL schematic representation of two differentiation blocks connected by a waveguide of length L_c with impedance Z_c and a propagation constant β. An incident time domain signal $f(t)$ is applied from the left-most waveguide. Due to the interference of reflections occurring between the two blocks (red arrows), the signal transmitted through the whole structure to the right-most waveguide may not necessarily represent the second order derivative of the incident signal. A feature that can be tackled by engineering the length of the connecting waveguide. **b**, Contour plots showing the analytical calculated transmission coefficient, magnitude (left) and phase (right), in the range 0.75 - $1.25 f_0$ for L_c ranging between $0.2\,\lambda_0$ and λ_0 ($\lambda_0 = 37.5$ mm, $f_0 = 8$ GHz). In these calculations the 2 differentiator blocks are made from parallel junctions with PEC terminated stubs of length $\lambda_0/2$. All waveguides (input, output, stubs, and connection waveguides) are filled with vacuum ($\varepsilon_r = 1, \mu_r = 1$) and have a characteristic impedance Z_0. **c**, and **d**, are horizontal and vertical profile plots, respectively, of the data presented in **b**. **c**, shows the transmission coefficient, magnitude (black) and phase (red), for fixed values of L_c. The left and right panels show transmission coefficient when L_c is the first two odd and even integer multiples of $\lambda_0/4$, respectively, with solid lines representing $\lambda_0/4$, $\lambda_0/2$ and dashed lines representing $3\lambda_0/4$ and λ_0. **d**, shows the transmission coefficient as in **c**, (magnitude, left, phase, right panels) now for a fixed frequency as L_c varies. The solid and dashed plots show the cases where $f = (1 - 0.125)f_0$ and $f = (1 + 0.125)f_0$ respectively.

To evaluate the performance of cascaded temporal differentiators, an analytical study of the impact of different connection lengths onto the transmission coefficient of a two-differentiator structure was performed. The Redheffer star product method (described in detail in the main text) was used to calculate the transmission and reflection coefficient of the combined structure. The two first order differentiator blocks where made with two closed stubs (stubs with length $\lambda_0/2$, for $\lambda_0 = 37.5$ mm) connected at a parallel junction. A waveguide of length L_c is used to connect the two temporal differentiators. All waveguides in this scenario have the same characteristic impedance Z_0 (representing the impedance of free space) and vacuum as a filling material ($\varepsilon_r = 1, \mu_r = 1$).

As is explained in the main text, the transmission and reflection of the individual isolated differentiators is described by a pair of scattering matrices, $\boldsymbol{A}^1$ and $\boldsymbol{A}^2$ for differentiator 1 and 2 respectively. The matrix elements of $\boldsymbol{A}^1$ and $\boldsymbol{A}^2$ are given by Eq. S12 with $A_{12}^1 = A_{21}^1 = A_{12}^2 = A_{21}^2 = T$ and $A_{11}^1 = A_{22}^1 = A_{11}^2 = A_{22}^2 = \Gamma_{M+1}$ calculated using Eq. S12a and Eq. S12b, respectively. To represent the propagation of signals between the differentiators, a phase change is applied to the matrix elements of one of the scattering matrices (in this case $\boldsymbol{A}^1$) such that $A_{11}^1 \rightarrow A_{11}^1 e^{-2i\varphi_c}$, $A_{22}^1 \rightarrow A_{22}^1 e^{-2i\varphi_c}$, $A_{12}^1 \rightarrow A_{12}^1 e^{-i\varphi_c}$ and $A_{21}^1 \rightarrow A_{21}^1 e^{-i\varphi_c}$, where $\varphi_c = \omega L_c/c$ is the electrical length of the connection waveguide. The overall scattering matrix ($\boldsymbol{A}^3$) of the system is then calculated using Eq. 10 from the main text (with $\boldsymbol{A} = \boldsymbol{S}$)and the transmission/reflection coefficients are extracted from the calculated A_{11}^3 and A_{21}^3 terms.

The impact of the connection length onto the transmission coefficient can be seen in Fig. S3b where the magnitude (left) and phase (right) of the calculated overall transmission coefficient is shown as a function of f and L_c. Here, f ranges from $0.75f_0$ to $1.25f_0$ for $f_0 = 8$ GHz, and L_c from $0.2\lambda_0$ to λ_0 with $\lambda_0 = 37.5$ mm. As can be clearly seen, for most of the connection lengths, the transmission coefficient is no-longer symmetrical around the minimum at f_0. This would negatively impact the performance of the cascaded temporal differentiator, due to the symmetry requirements of the ideal transfer function (U-shape). In fact, as expected from TL theory, only connection lengths which are a multiple of $\lambda_0/4$ preserve the symmetry around f_0. This is highlighted in Fig. S3c which shows the transmission coefficient when L_c is a multiple of $\lambda_0/4$. These results have been separated into odd (left, $L_c = \lambda_0/4, 3\lambda_0/4$) and even (right, $L_c = 2\lambda_0/4, 4\lambda_0/4$) integer multiples. In both cases the symmetry is preserved around f_0, but only in the odd integer multiple case does the order of the differentiator increase (from a linear V-shaped to a quadratic U-shaped transmission coefficient). This is due to the destructive interference of reflections when L_c is an odd integer multiple of $\lambda/4$, and the constructive interference of the reflections when L_c is instead an even integer multiple $\lambda_0/4$. The symmetry of the

transmission coefficient is also highlighted in Fig. S3d which shows the transmission coefficient, magnitude (left) and phase (right), at a pair of fixed frequencies, $f = (1 - 0.125)f_0$ (solid line) and $f = (1 + 0.125)f_0$ (dashed line), as L_c is varied (results extracted from Fig. S3b). As can be seen, the intersection points of these two functions indicate the values of L_c which preserve the symmetry of the transmission coefficient around f_0.

4. First order differentiator: bandwidth

To further evaluate the performance of the first order differentiator, a study of the effect of signal bandwidth on the quality of the differentiated signal was performed. As the transmission coefficient of an M-stub junction (Eq. 4 from the main text) is only linear locally around f_0 it is expected that the quality of the temporal derivative produced at the output of the network will deteriorate for higher bandwidth incident signals.

To investigate this, a differentiator was constructed in CST Studio Suite® from two PEC-terminated stubs connected at a parallel plate waveguide junction. All waveguides were filled with vacuum, had dimensions $w = h = 0.0267\lambda_0$ and a characteristic impedance of Z_0. Three stub lengths (L_s) were investigated corresponding to the lengths required for the first, second and third minimum in the transmission coefficient to appear at f_0. Analytically this occurred when $L_s = \lambda_0/2\,, \lambda_0$ and $3\lambda_0/2$ but due to the effects of imperfect splitting outlined in the main text, the final stub values used where $L_s = 0.5227\lambda_0, 1.0222\lambda_0$ and $1.5205\lambda_0$, respectively. A schematic representation of this setup (TL) is shown in Fig. S4a. The magnitude of the transmission coefficient for the three stub lengths is shown in Fig. S4b showing how the first, second and third minimum in the transmission coefficient appears at f_0, respectively for the three stub lengths. A range of temporal Gaussian input signals, modulated at $f_0 = 8$ GHz, with frequency domain full width half maximums (FWHMs) ranging from $0.05f_0$ to $0.4f_0$ is excited sequentially at the input waveguide of each structure. In each case the performance of the differentiator is evaluated by calculating the root mean squared error (RMSE) between the time domain signal observed at the output of the structure and the analytically calculated derivative of the incident signal, the results of which are shown in Fig. S4c for the three structures, respectively.

As expected, the error in the observed derivative increases for input pulses with a higher bandwidth. For the lowest value of FWHM, there is initially little difference between the error of the first and second minimum, as seen in Fig. S4c. This is because in both cases the frequency content of the Gaussian pulse is entirely contained within the linear region of the respective transmission coefficients. This, however, is not the case for the third minimum, as the transmission coefficient begins to curve closer to f_0 than the other two cases, and consequently there is a noticeable increase in the RMSE. Fig. S4d shows the frequency domain input (top) and output signals from all three structures when excited with the $0.4f_0$ FWHM Gaussian pulse. As can be seen, for all structures there is a good agreement between the numerical and analytical results in the region around f_0 (where all three transmission coefficients resemble a linear V-shape), however at higher and lower frequencies the results diverge. This is more pronounced for the second and third minimum as the region around f_0 where the transmission coefficient may be approximated as linear is smaller than the first minimum. In the case of the third minimum, it

can be observed how due to the narrower bandwidth of the transmission coefficient, frequency content of the incident Gaussian may "*leak*" into the adjacent minima, further increasing the error in the observed derivative. This is evident in Fig. S4d as the frequency domain output of the third structure has a minimum at $0.66f_0$ and $1.33f_0$, due to the impact of the adjacent minima in the transmission coefficient.

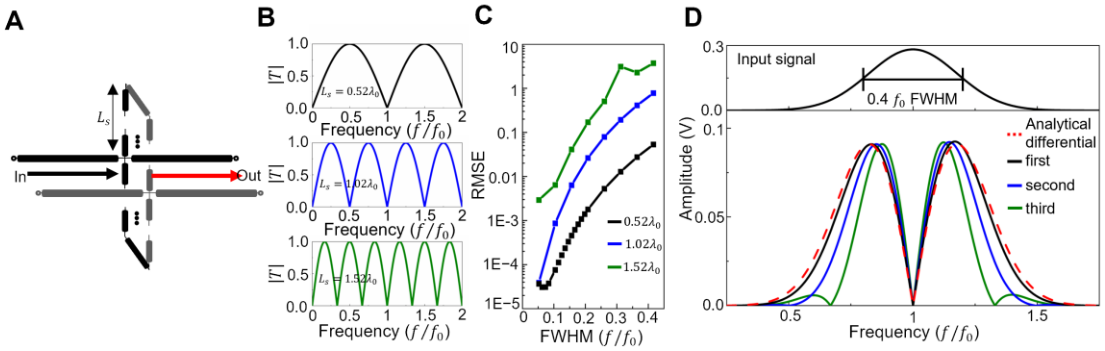

Fig S4| Bandwidth study for two-stub first order differentiators. a, TL schematic representation of a differentiator made from two closed stubs connected to an input and output waveguide at a parallel waveguide junction. All waveguides are filled with vacuum ($\varepsilon_r = 1, \mu_r = 1$) and have the same characteristic impedance Z_0. Both stubs have the same length L_s. **b**, Magnitude of the transmission coefficient of this structure for stub lengths $0.5227\lambda_0$, $1.0222\lambda_0$ and $1.5205\lambda_0$ (black, blue and green lines, respectively), corresponding to the lengths at which the first second and third transmission coefficient minimum appears at the frequency f_0 ($\lambda_0 =$ 37.5 mm, $f_0 = 8$ GHz). An incident Gaussian signal is applied at the left waveguide (black arrow) and the output signal is recorded at the right waveguide (red arrow). **c**, RMSE between the recorded output signal for the three lengths provided above (black, blue and green lines respectively), and the analytically calculated derivative of the incident Gaussian signal as a function of the FWHM of the incident Gaussian. **d**, Frequency domain results showing the recoded outputs (bottom panel) of the three structures (black, blue and green lines respectively) when an incident Gaussian (top panel) with a FWHM of $0.4f_0$ is applied at the input. This is compared with the analytical values for the derivative of the incident Gaussian in the frequency domain (red dashed line).
